# Reproducibility of global and segmental myocardial strain using cine DENSE at 3 T: a multicenter cardiovascular magnetic resonance study in healthy subjects and patients with heart disease

**DOI:** 10.1186/s12968-022-00851-7

**Published:** 2022-04-04

**Authors:** Daniel A. Auger, Sona. Ghadimi, Xiaoying Cai, Claire E. Reagan, Changyu Sun, Mohamad Abdi, Jie Jane Cao, Joshua Y. Cheng, Nora Ngai, Andrew D. Scott, Pedro F. Ferreira, John N. Oshinski, Nick Emamifar, Daniel B. Ennis, Michael Loecher, Zhan-Qiu Liu, Pierre Croisille, Magalie Viallon, Kenneth C. Bilchick, Frederick H. Epstein

**Affiliations:** 1 Department of Biomedical Engineering, University of Virginia, Box 800759, Charlottesville, VA 22908 USA; 2 Radiology and Medical Imaging, University of Virginia Health System, Charlottesville, VA USA; 3 St. Francis Hospital, The Heart Center, Long Island, NY USA; 4 Cardiovascular Magnetic Resonance Unit, The Royal Brompton Hospital and National Heart and Lung Institute, Imperial College London, London, UK; 5 Department of Radiology & Imaging Sciences and Biomedical Engineering, Emory University, Atlanta, Georgia; 6 Department of Radiology, Stanford University, Stanford, CA USA; 7 University of Lyon, UJM-Saint-Etienne, INSA, CNRS UMR 5520, INSERM U1206, CREATIS, Saint-Etienne, France; 8 Department of Radiology, University Hospital Saint-Etienne, Saint-Etienne, France; 9 Siemens Healthineers, Boston, Massachusetts USA

**Keywords:** DENSE, CMR, Myocardial strain imaging, Reproducibility

## Abstract

**Background:**

While multiple cardiovascular magnetic resonance (CMR) methods provide excellent reproducibility of global circumferential and global longitudinal strain, achieving highly reproducible segmental strain is more challenging. Previous single-center studies have demonstrated excellent reproducibility of displacement encoding with stimulated echoes (DENSE) segmental circumferential strain. The present study evaluated the reproducibility of DENSE for measurement of whole-slice or global circumferential (E_cc_), longitudinal (E_ll_) and radial (E_rr_) strain, torsion, and segmental E_cc_ at multiple centers.

**Methods:**

Six centers participated and a total of 81 subjects were studied, including 60 healthy subjects and 21 patients with various types of heart disease. CMR utilized 3 T scanners, and cine DENSE images were acquired in three short-axis planes and in the four-chamber long-axis view. During one imaging session, each subject underwent two separate DENSE scans to assess inter-scan reproducibility. Each subject was taken out of the scanner and repositioned between the scans. Intra-user, inter-user-same-site, inter-user-different-site, and inter-user-Human-Deep-Learning (DL) comparisons assessed the reproducibility of different users analyzing the same data. Inter-scan comparisons assessed the reproducibility of DENSE from scan to scan. The reproducibility of whole-slice or global E_cc_, E_ll_ and E_rr_, torsion, and segmental E_cc_ were quantified using Bland–Altman analysis, the coefficient of variation (CV), and the intraclass correlation coefficient (ICC). CV was considered excellent for CV ≤ 10%, good for 10% < CV ≤ 20%, fair for 20% < CV ≤ 40%, and poor for CV > 40. ICC values were considered excellent for ICC > 0.74, good for ICC 0.6 < ICC ≤ 0.74, fair for ICC 0.4 < ICC ≤ 0.59, poor for ICC < 0.4.

**Results:**

Based on CV and ICC, segmental E_cc_ provided excellent intra-user, inter-user-same-site, inter-user-different-site, inter-user-Human-DL reproducibility and good–excellent inter-scan reproducibility. Whole-slice E_cc_ and global E_ll_ provided excellent intra-user, inter-user-same-site, inter-user-different-site, inter-user-Human-DL and inter-scan reproducibility. The reproducibility of torsion was good–excellent for all comparisons. For whole-slice E_rr_, CV was in the fair-good range, and ICC was in the good–excellent range.

**Conclusions:**

Multicenter data show that 3 T CMR DENSE provides highly reproducible whole-slice and segmental E_cc_, global E_ll_, and torsion measurements in healthy subjects and heart disease patients.

## Background

Myocardial strain imaging for the assessment of contractile function has multiple applications. It is used for the detection of cardiotoxicity due to chemotherapy [1], in cardiac resynchronization therapy (CRT) as a predictor of response and outcomes [2, 3] and for detecting late-activating regions [4, 5], as a predictor of major adverse cardiac events after myocardial infarction [6], for the detection ischemia [7], and in other areas. For some applications, global strain assessments may be adequate; however, for others, such as detecting late-activating regions or for imaging regions of infarction or ischemia, segmental strain quantification is essential. Furthermore, reproducible techniques for segmental strain could potentially demonstrate its importance in certain applications where global strain is currently thought to be sufficient, such as cardiotoxicity where dysfunction may not be uniform throughout the myocardium, but instead may effect some regions more than others [8].

Strain reproducibility, i.e., the ability to obtain a consistent strain result when a measurement is repeated, is important for all of the applications discussed. The assessment of myocardial strain by cardiovascular magnetic resonance (CMR) takes multiple steps, including those that are related to image acquisition and those that occur during image analysis, and these steps may be influenced by practices and conditions that may vary between different users, different sites, and different imaging scans and sessions. For these reasons, reproducibility studies should try to account for as many of these variables as possible.

While a number of studies have shown high reproducibility of global or whole-slice strain for multiple CMR strain imaging methods including feature tracking [9, 10], conventional tissue tagging [10, 11], harmonic phase (HARP) imaging [12], strain-encoded imaging (SENC) [10, 13], and displacement encoding with stimulated echoes (DENSE) [11, 14, 15], achieving highly reproducible results for segmental strain is more challenging. For example, it has been shown that the reproducibility of segmental strain assessed using feature tracking is in the fair-poor range [16–20]. Previously, two single-center studies reported that DENSE at 1.5 T provides highly reproducible measurements of segmental end-systolic circumferential shortening (E_cc_) in healthy subjects [15, 21], and another 1.5 T study showed excellent intra-observer and inter-observer reproducibility of DENSE segmental E_cc_ in myocardial infarction patients [22]. The study by Lin et al. [21] in particular showed excellent reproducibility at one center of DENSE for different image acquisition sessions (inter-session reproducibility), the same user performing image analysis at different times (intra-user reproducibility), and for different users performing image analysis (inter-user reproducibility). Recent improvements to DENSE include the use of outer volume suppression and optimization of parameters for 3 T [23]. The present study sought to evaluate the reproducibility of 3 T DENSE at multiple centers, for multiple analysts (including human analysts at the same and different centers and analysis by fully-automated deep learning), and for successive scans in healthy subjects and in patients with heart disease, with a particular focus on segmental E_cc_.

## Materials and methods

### Study sites and subjects

Six centers participated in this study (St. Francis Hospital, New York, USA; the Royal Brompton Hospital, London, UK; Stanford University, Palo Alto, USA; University Hospital, Saint-Etienne, France; Emory University, Atlanta, USA; and the University of Virginia, Charlottesville, USA). All sites had prior experience with cardiac cine DENSE imaging. A total of 81 subjects participated, including 60 healthy subjects and 21 heart disease patients (Table 1). The patient group included subjects with (a) heart failure with left bundle branch block (n = 9), (b) ischemic heart disease (n = 3), (c) amyloidosis (n = 4), (d) cardiomyopathy (n = 3), atrial fibrillation (n = 1), and hypertension (n = 1). Only adults > 18 years were included, and subjects with CMR contraindications (e.g., implantable devices, cerebral aneurysm clips, cochlear implants etc.) were excluded. Additional exclusion criteria for the healthy control group included a history of cardiovascular disease, hypertension and smoking. All CMR studies were performed in accordance with each site’s protocols that were approved by their respective institutional review boards for research involving human subjects, and all subjects provided informed consent. All CMR was performed using 3 T CMR scanners (MAGNETOM Prisma or Skyra, Siemens Healthineers, Erlangen, Germany) with 4–32 channel phased-array radiofrequency coils.

**Table 1:** Description of Subjects

	Healthy Subjects (N = 60)	Cardiac Patients (N = 21)
Female	32	7
Heart failure—left bundle branch block	–	9
Ischemic heart disease	–	3
Amyloidosis	–	4
Cardiomyopathy with low EF	–	3
Atrial fibrillation	–	1
Hypertension		1
Age (years)	31 ± 10	62 ± 11*
Systolic blood pressure (mmHg)^1^	121 ± 14	127 ± 16
Diastolic blood pressure (mmHg)^1^	67 ± 7	77 ± 10*
Heart Rate^1^	68 ± 12	65 ± 7
LV end-diastolic volume (ml)^2^	142 ± 28	166 ± 57
LV end-systolic volume (ml)^2^	63 ± 14	82 ± 54
LV Ejection fraction (%)^2^	56 ± 5	54 ± 13
End-systolic E_cc_	− 0.18 ± 0.03	− 0.15 ± 0.05*
Basal	− 0.16 ± 0.02	− 0.14 ± 0.05*
Mid	− 0.18 ± 0.02	− 0.16 ± 0.05*
Apex	− 0.20 ± 0.03	− 0.16 ± 0.06*
End-systolic E_ll_	− 0.15 ± 0.02	− 0.14 ± 0.04
End-systolic E_rr_	0.35 ± 0.16	0.28 ± 0.15*
Basal	0.38 ± 0.16	0.33 ± 0.17
Mid	0.33 ± 0.14	0.27 ± 0.12
Apex	0.32 ± 0.17	0.24 ± 0.14
Torsion (°/cm)	2.79 ± 0.75	2.43 ± 1.56

^1^Blood pressure and heart rate results are based on 32 healthy subjects and 10 cardiac patients

^2^LV (left ventricular) volumes and ejection fraction are based on 20 patients and 19 healthy subjects

### CMR protocol

For each subject, cine DENSE images were acquired in three short-axis planes at basal, mid-ventricular and apical levels, and in the four-chamber long-axis view. Short-axis cine balanced steady-state free precession (bSSFP) images covering the left ventricle (LV) were also acquired for all subjects for the quantification of LV end diastolic volume (LVEDV), LV end systolic volume (LVESV), and LV ejection fraction (LVEF). Healthy subjects did not receive gadolinium. For patients undergoing clinical exams, DENSE imaging was completed prior to administration of gadolinium. A standardized spiral cine DENSE [24] acquisition protocol with outer volume suppression [23] was used with the following parameters: slice thickness = 8 mm, TR = 15 ms, TE = 1.26 ms, temporal resolution of 15 ms (with view sharing), pixel size = 3.4 × 3.4 mm^2^, FOV = 200 mm^2^, region of signal generation = 120 × 120 mm^2^, 2D in-plane displacement encoding using the simple three-point method [25, 26], displacement-encoding frequency = 0.1 cycles/mm, ramped flip angle with final flip angle of 15°, fat suppression, and a total of 4 spiral interleaves with 2 interleaves acquired per heartbeat. Each cine DENSE acquisition was performed during end-expiratory breath holding over 14 cardiac cycles, which consisted of 12 cardiac cycles for acquiring DENSE data and 2 cardiac cycles to acquire B0 field map data which was used to correct the spiral data for off-resonance, assuming a linear variation in B0 across the field of view. The cine bSSFP protocol was not standardized among the participating sites.

During one imaging session, each subject underwent two separate DENSE scans in order to assess inter-scan reproducibility. Each subject was taken out of the scanner and repositioned between the scans. We refer to the separate scans as Scan A and Scan B.

### Strain analysis of DENSE images

Well-established methods were used for strain analysis of DENSE images. Segmentation of the LV myocardium was performed semiautomatically using motion-guided segmentation [27], and manual correction was applied if needed. Next, a phase-unwrapping algorithm was applied to LV myocardial pixels, and, subsequently Lagrangian displacement and strain were calculated [28]. For short-axis images, the Lagrangian strain tensor was projected to the circumferential and radial directions relative to the LV center of mass to compute circumferential and radial strains (E_cc_ and E_rr_, respectively). For long-axis images, the Lagrangian strain tensor was projected to the longitudinal direction to compute longitudinal strain (E_ll_). For short-axis imaging, whole-slice and segmental strain analyses were performed, where segmental analysis used the American Heart Association 16-segment model [29]. For four-chamber long-axis images, the E_ll_ values from the two mid-ventricular segments (American Heart Association segments 9 and 12) were averaged to compute a single global E_ll_ value. Global torsion was derived by computing twist for the three short-axis slices and then computing the change in twist along the longitudinal direction. All sites were provided written, video and/or live instructions in order to standardize the strain analysis process.

### Reproducibility of strain analysis

To investigate the reproducibility of performing strain analysis, each site assigned two users to analyze DENSE images. The users had between 0 and 10 years of experience performing DENSE analysis. To assess intra-user reproducibility, the first user of each site analyzed Scan A data twice, with a 2–3 week interval between analysis sessions. To assess inter-user reproducibility within each site, User 1 and User 2 at each site analyzed Scan A datasets. To assess inter-scan reproducibility, User 1 analyzed Scan A and Scan B images at each site. One site, the University of Virginia, assigned one user (D.A.) to analyze the Scan A images of all other sites in order to assess the inter-site reproducibility of strain analysis. This user is referred to as User UVA. As fully automatic deep learning (DL) methods have recently been developed for whole-slice and segmental E_cc_ analysis of short-axis DENSE images [30], inter-user reproducibility was also assessed for DL vs. User 1 of all sites.

### Statistical analysis

Correlations between continuous variables were assessed using the squared Pearson’s correlation coefficient *r*^*2*^. Bland–Altman analysis was used to assess the agreement between different measurements. Reproducibility was quantified using the coefficient of variation (CV) and the intra-class correlation coefficient (ICC). CV was considered excellent for CV ≤ 10%, good for 10% < CV ≤ 20%, fair for 20% < CV ≤ 40%, and poor for CV > 40% [21]. ICC values were considered excellent for ICC > 0.74, good for ICC 0.6 < ICC ≤ 0.74, fair for ICC 0.4 < ICC ≤ 0.59, poor for ICC < 0.4 [13]. ICC and CV values are presented for whole-slice or global E_cc_, E_rr_, E_ll_, and torsion, and for segmental E_cc_. Student t-tests were two-tailed with a significance level of p < 0.05.

## Results

**Fig. 1:**
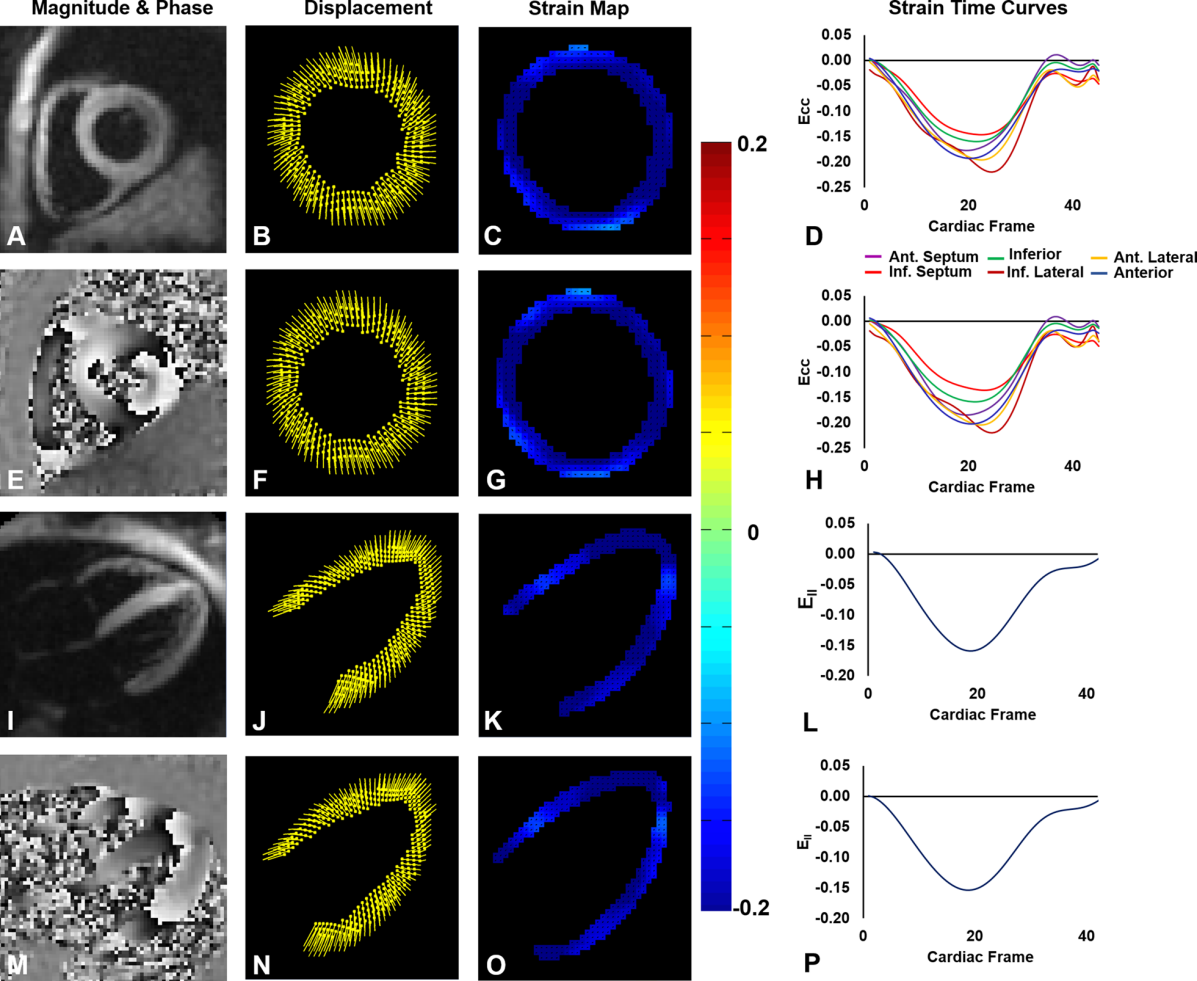
Demonstrations of intra-user and inter-user-same-site reproducibility. Example end-systolic short-axis displacement encoding with stimulated echoes (DENSE) magnitude (**A**) and phase (**E**) images of a healthy subject and the corresponding displacement maps (**B**, **F**), E_cc_ maps (**C**, **G**), and segmental circumferential strain (E_cc_)-time curves (**D**, **H**) resulting from analysis by the same user at two different times (**B**, **C**, **D** vs. **F**, **G**, **H**). Also shown are example end-systolic long-axis DENSE magnitude (**I**) and phase (**M**) images of a healthy subject and the corresponding displacement maps (**J**, **N**), E_ll_ maps (**K**, **O**), and global longitudinal strain (E_ll_)-time curves (L, P) resulting from analysis by two different users from the same site (**J**, **K**, **L** vs. N, **O**, **P**)

**Fig. 2:**
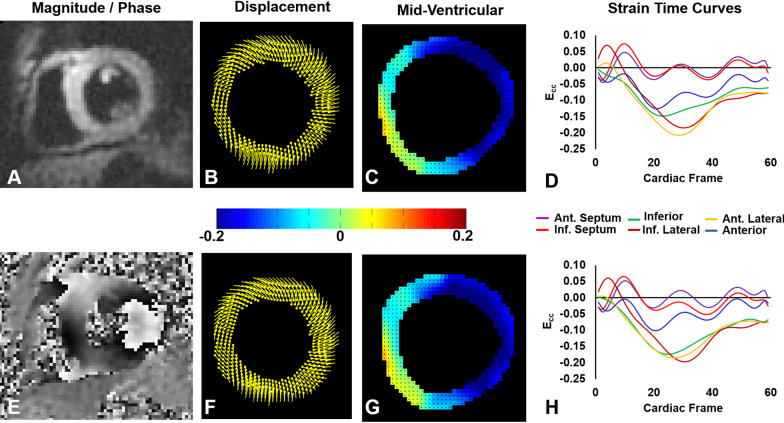
Demonstration of inter-user-different-site reproducibility. Example end-systolic short-axis DENSE magnitude (**A**) and phase (**E**) images of a patient with heart failure and left bundle branch block are shown as are the corresponding displacement maps (**B**, **F**), E_cc_ maps (**C**, **G**), and segmental E_cc_-time curves (**D**, **H**) resulting from analysis by users at two different sites (**B**, **C**, **D** vs. **F**, **G**, **H**)

Table 1 provides an overview of the study participants. From the 81 subjects, three short-axis slices were discarded due to poor image quality (phase signal-to-noise ratio [30] less than 12), resulting in 240 short-axis slices that underwent strain analysis and providing 240 strain values for whole-slice E_cc_ and E_rr_ reproducibility calculations, 78 values for torsion reproducibility calculations, and 1,278 strain values for segmental end-systolic E_cc_ reproducibility calculations. For long-axis imaging, eight slices were discarded due to poor image quality (phase signal-to-noise ratio less than 12 or a field of view that was smaller than the LV), resulting in 73 global E_ll_ values for reproducibility calculations. Example end-systolic short-axis and long-axis DENSE images from healthy subjects are shown in Fig. 1 along with displacement and strain maps and strain–time curves. This figure also shows displacement and strain data analyzed by the same user twice and by two different users at the same site. Example images and E_cc_ maps from a heart failure patient with left bundle branch block are shown in Fig. 2, as are E_cc_-time curves generated by two users from different sites, demonstrating inter-user-different-site reproducibility.

### Reproducibility of E_cc_

**Fig. 3:**
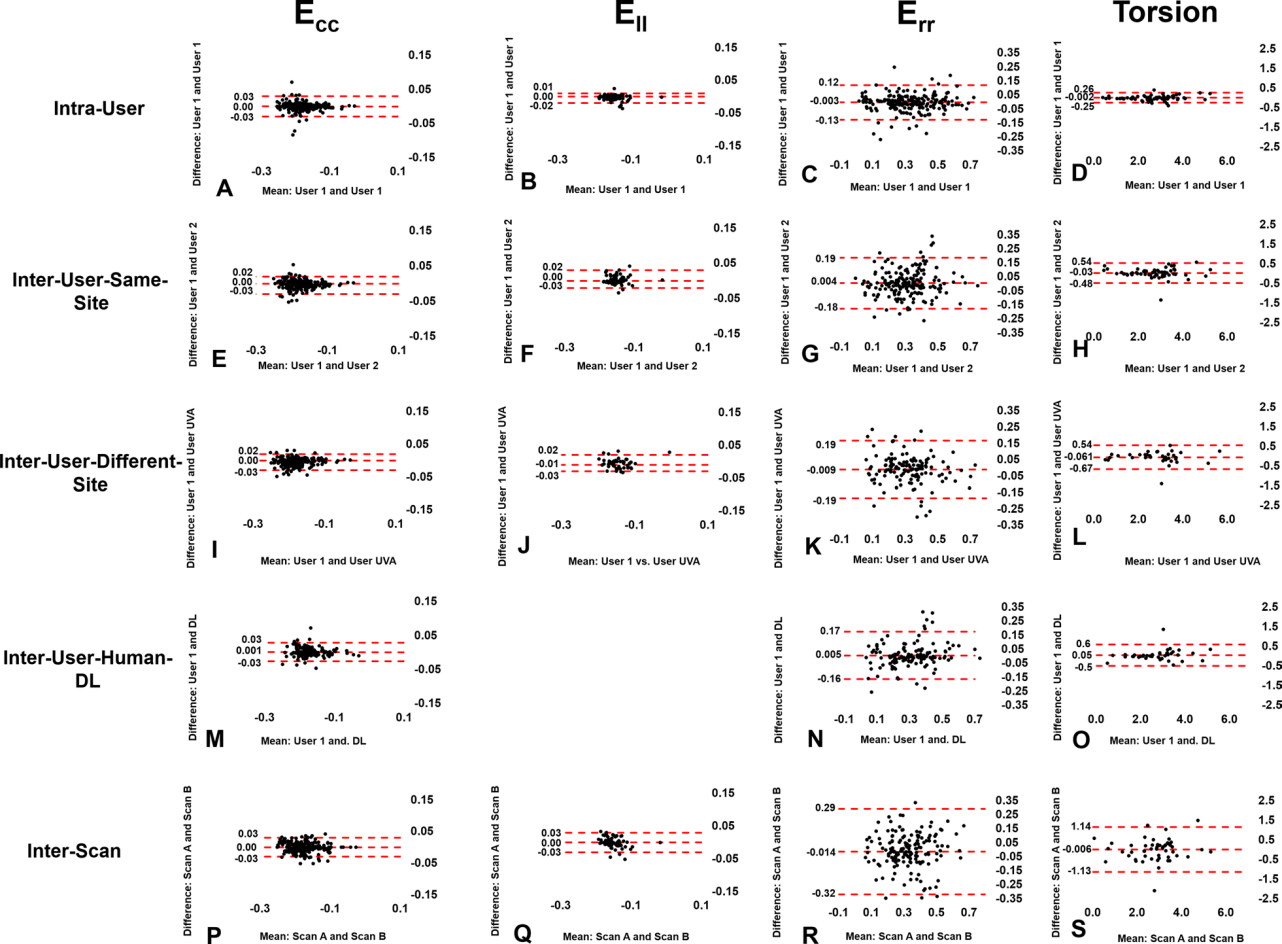
Bland–Altman plots for whole-slice or global E_cc_, E_ll_, radial strain (E_rr_) and torsion showing agreement of intra-user, inter-user-same-site, inter-user-different-site, inter-user-Human-DL and inter-scan comparisons

**Fig. 4:**
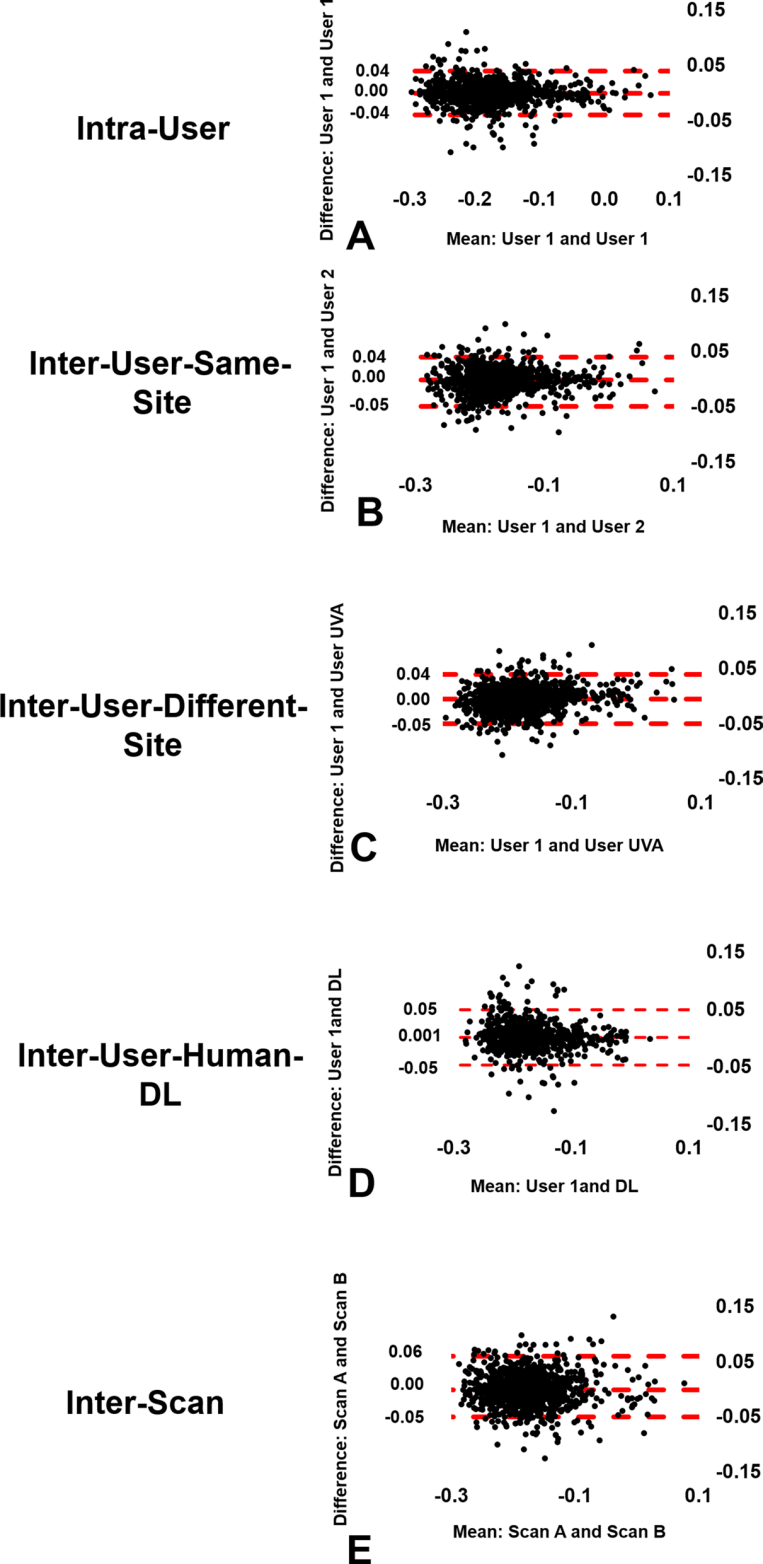
Bland–Altman plots for segmental E_cc_, showing agreement of intra-user, inter-user-same-site, inter-user-different-site, inter-user-Human-DL and inter-scan comparisons

**Fig. 5:**
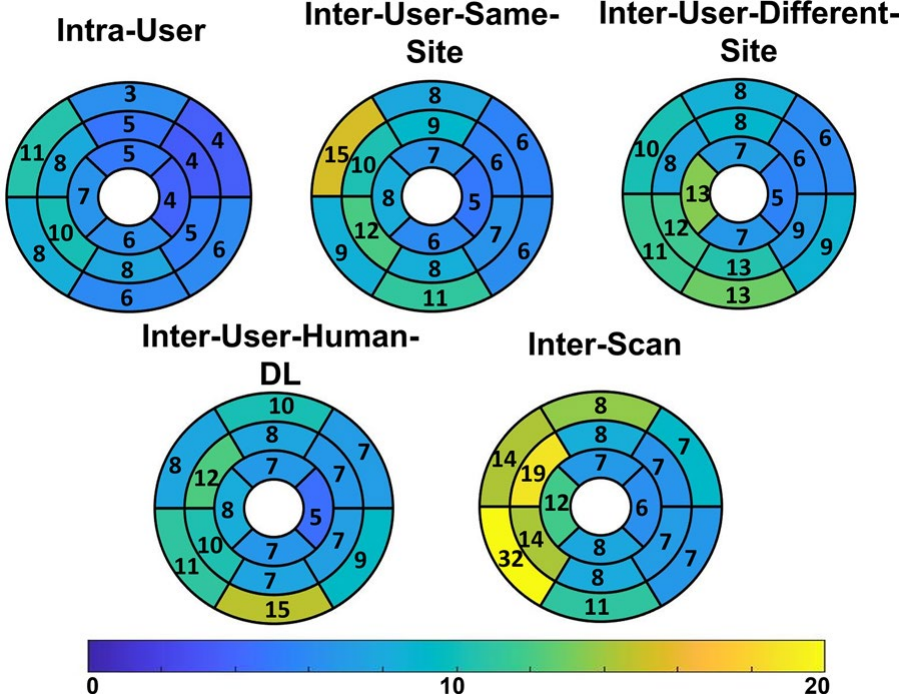
Bull’s eye plots of the coefficient of variation for segmental E_cc_ for intra-user, inter-user-same-site, inter-user-different-site, inter-user-human-DL and inter-scan comparisons

**Fig. 6:**
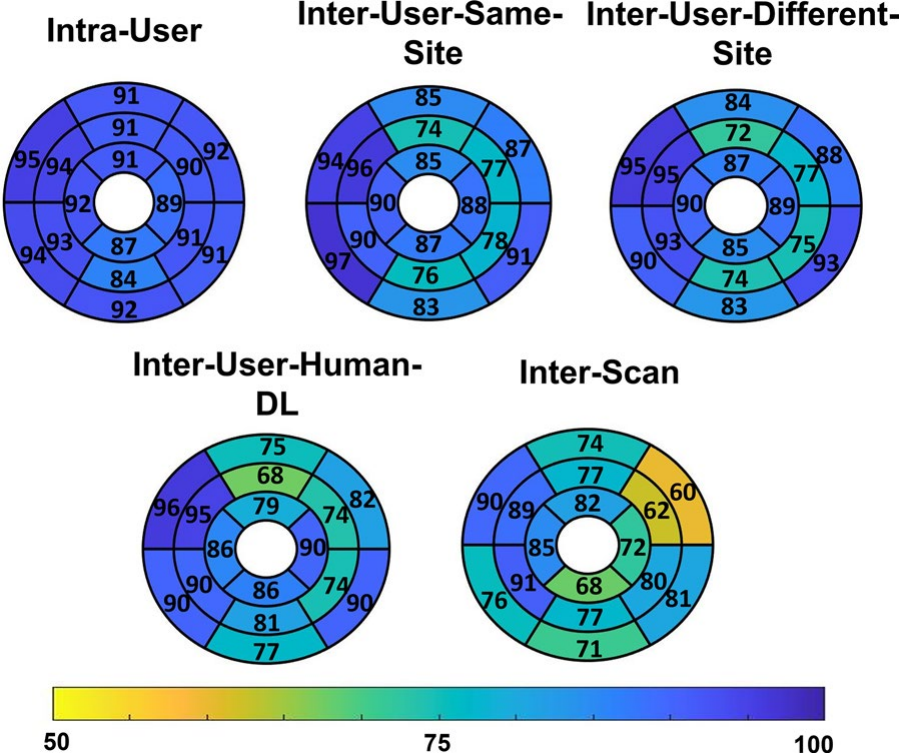
Bull’s eye plots of the intraclass correlation coefficient for segmental E_cc_ for intra-user, inter-user-same-site, inter-user-different-site, inter-user-human-DL and inter-scan comparisons

The global E_cc_ values, averaged over all three short-axis slices, were − 0.18 ± 0.03 and − 0.15 ± 0.05 (p < 0.05) for healthy subjects and for heart disease patients, respectively (see Table 1 for greater detail). For whole-slice E_cc_, Fig. 3 (column 1) shows Bland–Altman plots for intra-user, inter-user-same-site, inter-user-different-site, inter-user-Human-DL, and inter-scan comparisons, demonstrating narrow limits of agreement and small biases for all cases. For segmental E_cc_, Fig. 4 shows Bland–Altman plots for intra-user, inter-user-same-site, inter-user-different-site, inter-user-Human-DL, and inter-scan comparisons, demonstrating slightly higher but still narrow limits of agreement and small biases. As shown in Table 2, for whole-slice E_cc_ the mean coefficient of variation value was 5.0% or lower for intra-user, inter-user-same-site, inter-user-different-site, inter-user-Human-DL, and inter-scan comparisons, and the mean ICC was 0.85–0.93 for all comparisons. As also shown in Table 2, for segmental E_cc_ the coefficient of variation was 8.9% or lower for intra-user, inter-user-same-site, inter-user-different-site, and inter-user-Human-DL comparisons, and was 11.4 for the inter-scan case. The ICC was 0.86 or higher for all comparisons except inter-scan, where it was 0.77. For segmental E_cc_, bullseye plots of coefficient of variation and ICC for intra-user, inter-user-same-site, inter-user-different-site, inter-user-Human-DL, and inter-scan comparisons are shown in Figs. 5 and 6, respectively. These results indicate excellent intra-user, inter-user-same-site, inter-user-different-site, inter-user-Human-DL, and inter-scan reproducibility of whole-slice E_cc_ and excellent reproducibility of segmental E_cc_ for intra-user, inter-user-same-site, inter-user-different-site, and inter-user-Human-DL cases and good–excellent reproducibility of segmental E_cc_ for the inter-scan case, with coefficient of variation in the good range and ICC in the excellent range.

**Table 2:** Summary of correlation, Bland–Altman, CV and ICC results. Global E_cc_ and E_rr_ values are averaged over 3 slices (base, mid-ventricle and apex)

Group	Variability	r^2^	Bias	Limits (Lower:Upper)	CV	ICC	95% CI of ICC
**E**_**cc**_ *Global*	Intra-User	0.89	0.000	− 0.03:0.03	3.0 ± 0.3	0.93 ± 0.01	0.90–0.97
	Inter-User-Same-Site	0.89	0.010	− 0.03:0.02	3.7 ± 0.4	0.93 ± 0.03	0.85–1.0
	Inter-User-Different-Site	0.90	0.005	− 0.02:0.03	4.0 ± 0.5	0.93 ± 0.03	0.87–0.99
	Inter-User-Human-DL	0.88	0.001	− 0.03:0.03	3.8 ± 0.3	0.88 ± 0.04	0.82–0.92
	Inter-Scan	0.80	0.001	− 0.03:0.03	5.0 ± 0.3	0.85 ± 0.04	0.78–0.93
**E**_**ll**_ *Global*	Intra-User	0.90	0.002	− 0.01:0.02	2.9	0.94	–
	Inter-User	0.79	− 0.003	− 0.03:0.02	4.5	0.88	–
	Inter-Site	0.8	0.006	− 0.02:0.03	5.5	0.88	–
	Inter-Session	0.73	0.001	− 0.03:0.03	5.2	0.84	–
**E**_**rr**_ *Global*	Intra-User	0.85	0.003	− 0.13:0.12	24.6 ± 4.7	0.92 ± 0.05	0.88–0.95
	Inter-User-Same-Site	0.61	− 0.004	− 0.18:0.19	47 ± 16.5	0.75 ± 0.09	0.68–0.83
	Inter-User-Different-Site	0.67	− 0.009	− 0.19:0.17	34 ± 12.2	0.80 ± 0.04	0.73–0.86
	Inter-User-Human-DL	0.69	0.005	− 0.16:0.17	19.7 ± 6.0	0.82 ± 0.04	0.91–0.96
	Inter-Scan	0.19	0.014	− 0.32:0.29	33 ± 7.5	0.74 ± 0.07	0.66–0.81
**E**_**cc**_ *Segmental*	Intra-User	0.88	0.000	− 0.04:0.04	6.4 ± 2.1	0.91 ± 0.03	0.93–0.90
	Inter-User-Same-Site	0.84	0.003	− 0.05:0.04	8.3 ± 2.8	0.86 ± 0.07	0.9–0.82
	Inter-User-Different-Site	0.84	0.005	− 0.05:0.04	8.9 ± 2.5	0.86 ± 0.08	0.81–0.90
	Inter-User-Human-DL	0.79	0.001	− 0.05:0.05	8.7 ± 3.7	0.83 ± 0.05	0.79–0.88
	Inter-Scan	0.74	0.001	− 0.05:0.06	11.4 ± 6.5	0.77 ± 0.09	0.72–0.82
**Torsion** *Global*	Intra-User	0.98	0.002	− 0.25:0.26	2.48	0.99	–
	Inter-User-Same-Site	0.94	0.030	− 0.48:0.54	5.41	0.97	–
	Inter-User-Different-Site	0.93	− 0.061	− 0.67:0.54	5.83	0.96	–
	Inter-User-Human-DL	0.99	0.050	− 0.49:0.60	2.97	0.99	–
	Inter-Scan	0.72	0.006	− 1.13:1.14	21.2	0.86	–

CV, coefficient of variation; DL, deep learning; ICC, intra-class correlation coefficient; Ecc, circumferential strain; Ell longitudinal strain; Err radial strain

### Reproducibility of global E_ll_

The mean global E_ll_ values were − 0.15 ± 0.02 and − 0.14 ± 0.04 for healthy subjects and for heart disease patients, respectively. For global E_ll_, Fig. 3 (column 2) shows Bland–Altman plots for intra-user, inter-user-same-site, inter-user-different-site, and inter-scan comparisons, demonstrating narrow limits of agreement for all cases. As shown in Table 2, for global E_ll_ the coefficient of variation was 5.5% or lower for intra-user, inter-user-same-site, inter-user-different-site, and inter-scan comparisons, and the ICC was 0.84 or higher for all comparisons, indicating excellent intra-user, inter-user-same-site, inter-user-different-site, and inter-scan reproducibility of global E_ll_.

### Reproducibility of whole-slice E_rr_

The mean global E_rr_ values were 0.35 ± 0.16 and 0.28 ± 0.15 (p < 0.05) for healthy subjects and for heart disease patients, respectively. For whole-slice E_rr_, Fig. 3 (column 3) shows Bland–Altman plots for intra-user, inter-user-same-site, inter-user-different-site, inter-user-human-DL, and inter-scan comparisons, demonstrating wider limits of agreement for all cases as compared to the corresponding plots for E_cc_ and E_ll_. As shown in Table 2, for whole-slice E_rr_ the coefficient of variation is in the range of 19.7 – 47% (good—poor) for intra-user, inter-user-same-site, inter-user-different-site, inter-user-human-DL, and inter-scan comparisons, and ICC was in the range of 0.74 – 0.92 (good – excellent) for all comparisons.

### Reproducibility of torsion

The mean global torsion values were 2.79 ± 0.75 and 2.43 ± 1.56°/cm for healthy subjects and for heart disease patients, respectively. For global torsion, Fig. 3 (column 4) shows Bland–Altman plots for intra-user, inter-user-same-site, inter-user-different-site, inter-user-Human-DL, and inter-scan comparisons, demonstrating narrow limits of agreement for all cases. As shown in Table 2, for torsion the coefficient of variation is in the range of 2.5–21.2% (excellent – fair) for intra-user, inter-user-same-site, inter-user-different-site, inter-user-human-DL, and inter-scan comparisons, and ICC was in the range of 0.86–0.99 (excellent) for all comparisons.

## Discussion

### Major findings

For spiral cine DENSE at 3 T, segmental E_cc_ was shown to provide excellent intra-user, inter-user-same-site, inter-user-different-site, inter-user-human-DL reproducibility and good–excellent inter-scan reproducibility, with CV in the good range and ICC in the excellent range for this case. Whole-slice E_cc_ was shown to provide excellent intra-user, inter-user-same-site, inter-user-different-site, inter-user-human-DL and inter-scan reproducibility. Also, global E_ll_ was shown to provide excellent intra-user, inter-user-same-site, inter-user-different-site, inter-user-human-DL and inter-scan reproducibility, and the reproducibility of torsion was good–excellent for all comparisons. For whole-slice E_rr_, CV was typically in the fair-good range, and ICC was in the good–excellent range. This result showing worse reproducibility of E_rr_ compared to the reproducibility of E_cc_ and E_ll_ is typical of all CMR strain methods that compute E_rr_ from short-axis images. This occurs because there are just a few pixels that span the LV wall in the radial direction, which presents challenges for the computation of strain in the radial direction. Due to it’s lower reproducibility, clinical decision making should rely less on radial strain, and due to their higher reproducibility, more on circumferential and longitudinal strain.

We generally found that intra-user reproducibility was best, followed by inter-user-same-site and inter-user-different-site, and then by inter-scan. These results are not surprising, as differences in performing image analysis between users may be greater than differences between the same user at different points in time, and there may be greater differences between different users at different sites compared to different users at a common site. Inter-scan differences may reflect differences in slice position or other factors that may differ between scans, leading to lower reproducibility than cases where the same images were analyzed at different times or by different users. With regard to the fully-automated DL method, its reproducibility was generally very similar to that of inter-user-same-site, which is consistent with and extends the results of the recent study that developed these methods [30]. For segmental E_cc_, we observed that lower ICC values were seen in the lateral wall, whereas higher CV values were seen in the septum. This occurred because to get a high segmental ICC value, it is important to have a fairly wide range of the strain data in that segment. For the patients in our study, most of the segmental dysfunction occurred in the septal segments, leading to a wide range of E_cc_ values in the septum but a very narrow range in the lateral wall. This spatial distribution of segmental dysfunction also explains why CV values were higher in the septum. Since the computation of CV includes dividing by the mean strain, higher CVs occurred in in the septal segments, where some end-systolic E_cc_ values were near zero. Specifically, with regard to computation of CV for any heart segment, we consider that there are two strain observations. First, we have the strain values for all myocardial points in a specific segment provided by one observation. Second, we have the strain values for the same myocardial points in same segment provided by another observation. Then, the mean strain is the average of the strains from the two different observations for each myocardial point. The reported CV for each segment is the mean coefficient of variation from all points within that segment.

Many cardiac imaging modalities report high reproducibility of global strain, including speckle-tracking echocardiography (STE) [31] and computed tomography based feature tracing (CT-FT) [32–34]. With regard to the reproducibility of segmental strain, the present DENSE circumferential strain data compare favorably to STE and CT-FT. While there is a limited amount of published data, for STE a few studies investigating the interobserver reproducibility of various segmental strains (longitudinal and circumferential) report ICC values in the range of 0.77 – 0.82 [35, 36], whereas the present study shows an ICC of 0.86 for DENSE E_cc_. For Bland–Altman limits of agreement for segmental strain, the values for STE are approximately twice as wide as for cine DENSE [36]. General recommendations for STE are that, while global strain is highly reproducible, segmental strain measurements have a higher degree of measurement variability and caution should be applied for clinical use [31]. For CT-FT, global strain also has high intraobserver and interobserver reproducibility [32–34]; however, the reproducibility of segmental strain is low, with ICC values less than 0.75 in most segments [34].

The present results contribute to a thorough sequence of studies validating DENSE for the measurement of displacement and strain. Spottiswoode et al. validated DENSE measurements of displacement in a rotating phantom for displacement values on the order of 2–20 mm [28], and Nwotchouang et al. recently validated DENSE measurements of displacement in a phantom designed for much smaller displacements, on the order of 20–200um [37]. Cowan et al. carefully validated DENSE measurements of strain using a deformable phantom, showing accuracy of strain that was similar to conventional tagging. The same study showed better reproducibility in healthy subjects of whole-slice E_cc_ and E_rr_ for DENSE than myocardial tagging [11]. Lin et al. showed the high reproducibility of DENSE for segmental E_cc_ at a single center, and these results included inter-session reproducibility with imaging sessions separated by days (not just minutes as in the present study). Verzhbinsky et al. used simulated phantom data to evaluate the methods used to compute strain from DENSE phase data and rigorously demonstrated their validity and accuracy [38]. Carruth et al. recently extended the findings of high reproducibility of DENSE strain to the right ventricle [14]. The demonstrated accuracy and reproducibility of DENSE may explain why E_cc_ measured by DENSE outperforms E_cc_ measured by feature tracking in clinical applications such as predicting CRT response [3] and predicting post-infarct LV remodeling and cardiac events [6].

Only 1.2% of short-axis slices and 9.9% of long-axis slices were not suitable for strain analysis. All sites had less prior experience with long-axis compared to short-axis DENSE utilizing outer volume suppression and a reduced field of view, which led to the higher percentage of poor image quality for long-axis imaging. Specifically, mistakes were made when using outer volume suppression for long-axis imaging such that the region of signal generation did not cover the entire LV. With greater experience, these mistakes were readily avoided.

## Limitations

Our study investigated inter-scan reproducibility where subjects were removed from the scanner, repositioned, and rescanned, but didn’t include inter-session reproducibility where subjects underwent DENSE CMR on different days. This limitation occurred because the study design involved adding DENSE scans to clinical patient scans. With the reliance on clinical scans, it was not possible to schedule additional sessions for the evaluation of inter-session reproducibility. The present study didn’t investigate the reproducibility of DENSE at 1.5 T; however, the single-center study by Lin et al. was performed at 1.5 T [21]. Only one vendor was included because the cine DENSE sequence is only available for Siemens CMR scanners. While we evaluated whole-slice E_rr_, we did not seek to show good-to-excellent reproducibility of segmental E_rr_ because prior data from Lin et al. strongly suggest that the reproducibility of segmental E_rr_ would be poor-to-fair [21]. We did not investigate the reproducibility of segmental E_ll_ for DENSE because we have less experience with and less standardization of methods for long-axis DENSE imaging and image analysis.

## Conclusions

In a multi-center study, 3 T CMR DENSE was shown to provide highly reproducible whole-slice and segmental E_cc_, global E_ll_, and torsion myocardial strain data in healthy subjects and patients with heart disease with regard to intra-user, inter-user-same-site, inter-user-different-site, inter-user-human-DL and inter-scan measurements. Fully-automated DL image analysis methods applied to short-axis DENSE images provide excellent reproducibility, equivalent to that of an expert user for whole-slice and segmental E_cc_ and for torsion. These findings may facilitate future clinical applications that would benefit from reproducible segmental strain imaging.

## Data Availability

Not applicable.

## Abbreviations

bSSFP: Balanced steady state free precession
CMR: Cardiovascular magnetic resonance
CRT: Cardiac resynchronization therapy
CT-FT: Computed tomography feature tracking
CV: Coefficient of variation
DENSE: Displacement encoding with stimulated echoes
DL: Deep learning
E_cc_: Circumferential shortening
E_ll_: Longitudinal strain
E_rr_: Radial strain
HARP: Harmonic phase
LV: Left ventricle/left ventricular
LVEDV: Left ventricular end-diastolic volume
LVEF: Left ventricular ejection fraction
LVESV: Left ventricular end-systolic volume
SENC: Strain-encoded imaging
STE: Speckle tracking echocardiography

## References

[1] Thavendiranathan P, Poulin F, Lim K-D, Plana JC, Woo A, Marwick TH (2014). Use of myocardial strain imaging by echocardiography for the early detection of cardiotoxicity in patients during and after cancer chemotherapy: a systematic review. J Am Coll Cardiol.

[2] Bilchick KC, Kuruvilla S, Hamirani YS (2014). Impact of mechanical activation, scar, and electrical timing on cardiac resynchronization therapy response and clinical outcomes. J Am Coll Cardiol.

[3] Bilchick KC, Auger DA, Abdishektaei M (2020). CMR DENSE and the Seattle heart failure model inform survival and arrhythmia risk after CRT. Cardiovasc Imaging.

[4] Khan FZ, Virdee MS, Palmer CR (2012). Targeted left ventricular lead placement to guide cardiac resynchronization therapy: the TARGET study: a randomized, controlled trial. J Am Coll Cardiol.

[5] Auger DA, Bilchick KC, Gonzalez JA (2017). Imaging left-ventricular mechanical activation in heart failure patients using cine DENSE MRI: Validation and implications for cardiac resynchronization therapy. J Magn Reson Imaging.

[6] Mangion K, Carrick D, Carberry J (2019). Circumferential strain predicts major adverse cardiovascular events following an acute ST-segment–elevation myocardial infarction. Radiology.

[7] Korosoglou G, Lehrke S, Wochele A (2010). Strain-encoded CMR for the detection of inducible ischemia during intermediate stress. JACC.

[8] Steen H, Montenbruck M, Gerzak B (2020). Cardiotoxicity during cancer treatment causes more regional than global dysfunction: the PREFECT Study. J Am Coll Cardiol.

[9] Gertz RJ, Lange T, Kowallick JT (2018). Inter-vendor reproducibility of left and right ventricular cardiovascular magnetic resonance myocardial feature-tracking. PLoS ONE.

[10] Bucius P, Erley J, Tanacli R (2020). Comparison of feature tracking, fast-SENC, and myocardial tagging for global and segmental left ventricular strain. ESC Heart Failure.

[11] Young AA, Li B, Kirton RS, Cowan BR (2012). Generalized spatiotemporal myocardial strain analysis for DENSE and SPAMM imaging. Magn Reson Med.

[12] Donekal S, Ambale-Venkatesh B, Berkowitz S (2013). Inter-study reproducibility of cardiovascular magnetic resonance tagging. J Cardiovasc Magn Reson.

[13] Giusca S, Korosoglou G, Zieschang V (2018). Reproducibility study on myocardial strain assessment using fast-SENC cardiac magnetic resonance imaging. Sci Rep.

[14] Carruth ED, Fielden SW, Nevius CD, Fornwalt BK, Haggerty CM. 3D-Encoded DENSE MRI with Zonal Excitation for Quantifying Biventricular Myocardial Strain During a Breath-Hold. Cardiovascular Engineering and Technology 2021:1–9. 10.1007/s13239-021-00561-8 PMC8714695 34244904

[15] Kar J, Knutsen AK, Cupps BP, Pasque MK (2014). A validation of two-dimensional in vivo regional strain computed from displacement encoding with stimulated echoes (DENSE), in reference to tagged magnetic resonance imaging and studies in repeatability. Ann Biomed Eng.

[16] Mangion K, Burke NM, McComb C, Carrick D, Woodward R, Berry C (2019). Feature-tracking myocardial strain in healthy adults-a magnetic resonance study at 3.0 tesla. Sci Rep.

[17] Wu L, Germans T, Güçlü A, Heymans MW, Allaart CP, van Rossum AC (2014). Feature tracking compared with tissue tagging measurements of segmental strain by cardiovascular magnetic resonance. J Cardiovasc Magn Reson.

[18] Lim C, Blaszczyk E, Riazy L (2021). Quantification of myocardial strain assessed by cardiovascular magnetic resonance feature tracking in healthy subjects—influence of segmentation and analysis software. Eur Radiol.

[19] Schuster A, Paul M, Bettencourt N (2013). Cardiovascular magnetic resonance myocardial feature tracking for quantitative viability assessment in ischemic cardiomyopathy. Int J Cardiol.

[20] Cowan BR, Peereboom SM, Greiser A, Guehring J, Young AA (2015). Image feature determinants of global and segmental circumferential ventricular strain from cine CMR. JACC.

[21] Lin K, Meng L, Collins JD, Chowdhary V, Markl M, Carr JC (2017). Reproducibility of cine displacement encoding with stimulated echoes (DENSE) in human subjects. Magn Reson Imaging.

[22] Mangion K, Carrick D, Clerfond G (2019). Predictors of segmental myocardial functional recovery in patients after an acute ST-Elevation myocardial infarction. Eur J Radiol.

[23] Tayal U, Wage R, Ferreira PF (2019). The feasibility of a novel limited field of view spiral cine DENSE sequence to assess myocardial strain in dilated cardiomyopathy. Magn Reson Mater Phys, Biol Med.

[24] Zhong X, Spottiswoode BS, Meyer CH, Kramer CM, Epstein FH (2010). Imaging three-dimensional myocardial mechanics using navigator-gated volumetric spiral cine DENSE MRI. Magn Reson Med.

[25] Zhong X, Helm PA, Epstein FH (2009). Balanced multipoint displacement encoding for DENSE MRI. Magn Reson Med.

[26] Kim D, Gilson WD, Kramer CM, Epstein FH (2004). Myocardial tissue tracking with two-dimensional cine displacement-encoded MR imaging: development and initial evaluation. Radiology.

[27] Spottiswoode BS, Zhong X, Lorenz CH, Mayosi BM, Meintjes EM, Epstein FH (2009). Motion-guided segmentation for cine DENSE MRI. Med Image Anal.

[28] Spottiswoode BS, Zhong X, Hess AT (2006). Tracking myocardial motion from cine DENSE images using spatiotemporal phase unwrapping and temporal fitting. IEEE Trans Med Imaging.

[29] American Heart Association Writing Group on Myocardial Segmentation and Registration for Cardiac Imaging:, Cerqueira MD, et al. Standardized myocardial segmentation and nomenclature for tomographic imaging of the heart: a statement for healthcare professionals from the Cardiac Imaging Committee of the Council on Clinical Cardiology of the American Heart Association. Circulation 2002;105:539–542. 10.1161/hc0402.102975 11815441

[30] Ghadimi S, Auger DA, Feng X (2021). Fully-automated global and segmental strain analysis of DENSE cardiovascular magnetic resonance using deep learning for segmentation and phase unwrapping. J Cardiovasc Magn Reson.

[31] Mirea O, Pagourelias ED, Duchenne J (2018). Variability and reproducibility of segmental longitudinal strain measurement: a report from the EACVI-ASE strain standardization task force. JACC.

[32] Miskinyte E, Bucius P, Erley J (2019). Assessment of global longitudinal and circumferential strain using computed tomography feature tracking: intra-individual comparison with CMR feature tracking and myocardial tagging in patients with severe aortic stenosis. J Clin Med.

[33] Vach M, Vogelhuber J, Weber M (2021). Feasibility of CT-derived myocardial strain measurement in patients with advanced cardiac valve disease. Sci Rep.

[34] Chen J, Zhang L-Y, Liu Y (2022). Left ventricular strain derived from computed tomography feature tracking: determinants of failure and reproducibility. Eur J Radiol.

[35] Sjøli B, Ørn S, Grenne B, Ihlen H, Edvardsen T, Brunvand H (2009). Diagnostic capability and reproducibility of strain by Doppler and by speckle tracking in patients with acute myocardial infarction. JACC.

[36] Yamada A, Luis SA, Sathianathan D (2014). Reproducibility of regional and global longitudinal strains derived from two-dimensional speckle-tracking and doppler tissue imaging between expert and novice readers during quantitative dobutamine stress echocardiography. J Am Soc Echocardiogr.

[37] Nwotchouang BST, Eppelheimer MS, Biswas D (2021). Accuracy of cardiac-induced brain motion measurement using displacement-encoding with stimulated echoes (DENSE) magnetic resonance imaging (MRI): a phantom study. Magn Reson Med.

[38] Verzhbinsky IA, Perotti LE, Moulin K, Cork TE, Loecher M, Ennis DB (2019). Estimating aggregate cardiomyocyte strain using In~ Vivo diffusion and displacement encoded MRI. IEEE Trans Med Imaging.

